# The association between photoreceptor layer thickness measured by optical coherence tomography and visual sensitivity in glaucomatous eyes

**DOI:** 10.1371/journal.pone.0184064

**Published:** 2017-10-12

**Authors:** Ryo Asaoka, Hiroshi Murata, Mieko Yanagisawa, Yuri Fujino, Masato Matsuura, Tatsuya Inoue, Kenji Inoue, Junkichi Yamagami

**Affiliations:** 1 Department of Ophthalmology, The University of Tokyo, Japan; 2 Inouye Eye Hospital, Tokyo, Japan; 3 JR Tokyo General Hospital, Tokyo, Japan; Massachusetts Eye & Ear Infirmary, Harvard Medical School, UNITED STATES

## Abstract

**Purpose:**

To assess the thickness of the photoreceptor layer in the macular region in glaucomatous eyes.

**Method:**

Humphrey 10–2 visual field (VF) testing was carried out and mean threshold (mTH) was calculated in 118 eyes from 118 patients with open angle glaucoma. Macular optical coherence tomography (OCT) measurements (RS 3000, Nidek Co.ltd., Aichi, Japan) were also carried out in all eyes. Thickness measurements were recorded in the outer segment and retinal pigment epithelium (OS+RPE), the nerve fiber layer (NFL), the ganglion cell layer and inner plexiform layer (GCL+IPL), the inner nuclear layer and outer plexiform layer (INL+OPL) and the outer nuclear layer and inner segment (ONL+IS). The relationship between mTH and the thickness of these five different layers was investigated. Additionally, the influence of OS+RPE on mTH was investigated using partial correlation eliminating the effect of other variables of NFL, GCL+IPL, INL+OPL, ONL+IS, age, gender and axial length.

**Results:**

The thickness of the OS+RPE layer was significantly decreased with the decrease of mTH (coefficient = 0.63 p <0.001). Partial correlation analysis suggested OS+RPE thickness is significantly (coefficient = 0.31, p <0.001) related to mTH, independent from NFL, GCL+IPL, INL+OPL, ONL+IS, age, gender and axial length.

**Conclusions:**

The thickness of the RPE+OS layer appears to be related to visual sensitivity in glaucoma.

## Introduction

Glaucoma is the leading cause of irreversible blindness in the world.[1, 2] The disease is a progressive optic neuropathy that can result in irrevocable visual field (VF) damage. The most common type of glaucoma is primary open-angle glaucoma (POAG) which affects more than 45 million people, with prevalence rates from 0.5% to 8.8%, depending on region.[3–22] Glaucoma is characterized by progressive degeneration of retinal ganglion cells (GC)s.[23, 24] The axons of retinal ganglion cells comprise the retinal nerve fiber (NF), and thus a thinning of the retina has been reported to occur in the inner layer of the retina where these NF layers locate.[23] Detailed thickness measurements of the retinal layer have become possible with the development of optical coherence tomography (OCT)[25–27] which uses low-coherence interferometry to produce a two-dimensional image of optical scattering from internal tissues.[28] While the importance of the inner retinal layer in glaucoma is well-established, the involvement of the photoreceptor (PhR) layer is controversial. Previous histological[29] and also electroretinography[30] studies have suggested the involvement of PhR cells in glaucoma, however, recent studies investigating OCT-measured thickness of the PhR layer have failed to show a decline in thickness of the PhR layer with the advancement of glaucoma.[31–33] In fact, one study showed the layer may be thicker in glaucoma patients owing to a swelling effect.[34] A similar involvement of PhR in optic neuritis has been reported in eyes with the disease.[35] Meanwhile, the effect of the PhR layer thickness on the visual sensitivity in glaucoma has not been investigated.

There are some limitations associated with the previous studies that investigated OCT-measured thickness of the outer retina in glaucoma patients.[31–33] First, there were a limited number of eyes studied (between 70 and 149 eyes including normative and glaucomatous eyes). Second the VF strategy deployed was either the 24–2 or 30–2 test pattern, measured with the Humphrey Field Analyzer (HFA, Carl Zeiss Meditec, Dublin, CA); in these tests VF sensitivity is measured across the central 30 degrees at a regular interval of six degrees. However, the OCT macular image corresponds to only the central ten degrees of the retina,[36, 37] and, indeed, the association between VF sensitivity in the central 10 degrees and central 30 degrees is weak.[38] Hence it is not entirely appropriate to measure the relationship between macular PhR layer thickness and VF sensitivity using a 24 degrees or 30 degrees VF test pattern. Furthermore, the association between the thicknesses of other intermediate layers, such as the inner nuclear layer (INL), the outer plexiform layer (OPL) and the outer nuclear layer (ONL), and the visual sensitivity in glaucomatous eyes has not been studied in detail.

In the current study, Humphrey 10–2 VF tests and OCT thickness measurements of the macular PhR layer were collected for the purpose of assessing the association between the thicknesses of the outer segment and retinal pigment epithelium (OS+RPE), the nerve fiber layer (NFL), the ganglion cell layer and inner plexiform layer (GCL+IPL), the inner nuclear layer and outer plexiform layer (INL+OPL) and the outer nuclear layer and inner segment (ONL+IS), and visual sensitivity in glaucoma.

## Materials and methods

The study was approved by the Research Ethics Committee of the Graduate School of Medicine and Faculty of Medicine at the University of Tokyo, Inoue Eye Hospital and JR Tokyo Sogo Hospital. Written informed consent was given by patients for their information to be stored in the hospital database and used for research. This study was performed according to the tenets of the Declaration of Helsinki.

### Subjects

The studied subjects comprised 118 glaucomatous (open angle glaucoma: OAG) eyes from 118 patients. All subjects underwent complete ophthalmic examinations, including biomicroscopy, gonioscopy, intraocular pressure measurement, funduscopy, refraction, best-corrected visual acuity measurements and axial length (AL) measurements, as well as OCT imaging and visual field (VF) testing. All study participants were enrolled between the period of April 2013 and June 2016 at either the University of Tokyo Hospital, JR Tokyo General Hospital or Inoue Eye Hospital; all of the patients satisfied the criteria and informed consent was obtained during the period of between January 2013 and June 2014 (Tokyo University Hospital), January 2014 and December 2015 (JR Tokyo General Hospital) and January 2014 and December 2015 (Inoue Eye Hospital) were enrolled in the study.

A diagnosis of glaucoma was made when reproducible glaucomatous changes in the optic nerve head, with or without retinal nerve fiber layer damage, was confirmed by a panel of glaucoma specialists (RA, HM, KI, JY), irrespective of the presence of glaucomatous VF change. This diagnostic method was used so that patients with a large range of glaucomatous damage were entered into the study, including those without measurable VF damage. If the diagnostic decisions of the examiners were not in agreement, a consensus was reached by group review and discussion. Subjects aged 20 years or older and eyes with visual acuity ≤0.5 LogMAR and axial length between 24.0 and 26.0 mm were included. People with other systemic or ocular disorders were carefully excluded; in particular, fundus photography and the photoreceptor inner segment/outer segment (IS/OS) line and the retinal pigment epithelium (RPE) on the OCT image were carefully reviewed by a specialist in macular diseases (TI) so that subjects with other retinal disease could be excluded from the study.

### VF measurement

VF testing was performed, within 3 months of the OCT examination, using the HFA with the SITA Standard strategy and the Goldmann size III target. Normative and glaucomatous eyes were measured with the 10–2 program. Near refractive correction was used as necessary. All of the participants had previous experience in VF examinations and unreliable VFs defined as fixation losses greater than 25%, or false-positive responses greater than 15% were excluded,[39] following the manufacturer’s recommendation. The mean of the whole field’s threshold values (mTH) was calculated and used in subsequent analyses. mTH is a mean of all threshold values. The mean deviation (MD) is similarly calculated from total deviation values, however it is weighted toward the center of visual field. This weighting is not appropriate in the current study, because the purpose of the current study was to investigate the relationship between retinal layer thicknesses on OCT and visual function, and thus we used the mTH value in the current study.

### OCT data acquisition

Spectral domain (SD)-OCT data were obtained using the RS 3000 (Nidek Co.ltd., Aichi, Japan). All SD-OCT measurements were performed after pupil dilation with 1% tropicamide and OCT imaging was performed using the raster-scan protocol. Data obtained during apparent eye movements or influenced by involuntary blinking or saccade, or, those with a Signal Strength index < 7 were excluded, as recommended by the manufacturer.

### Analysis of OCT data

**Fig 1:**
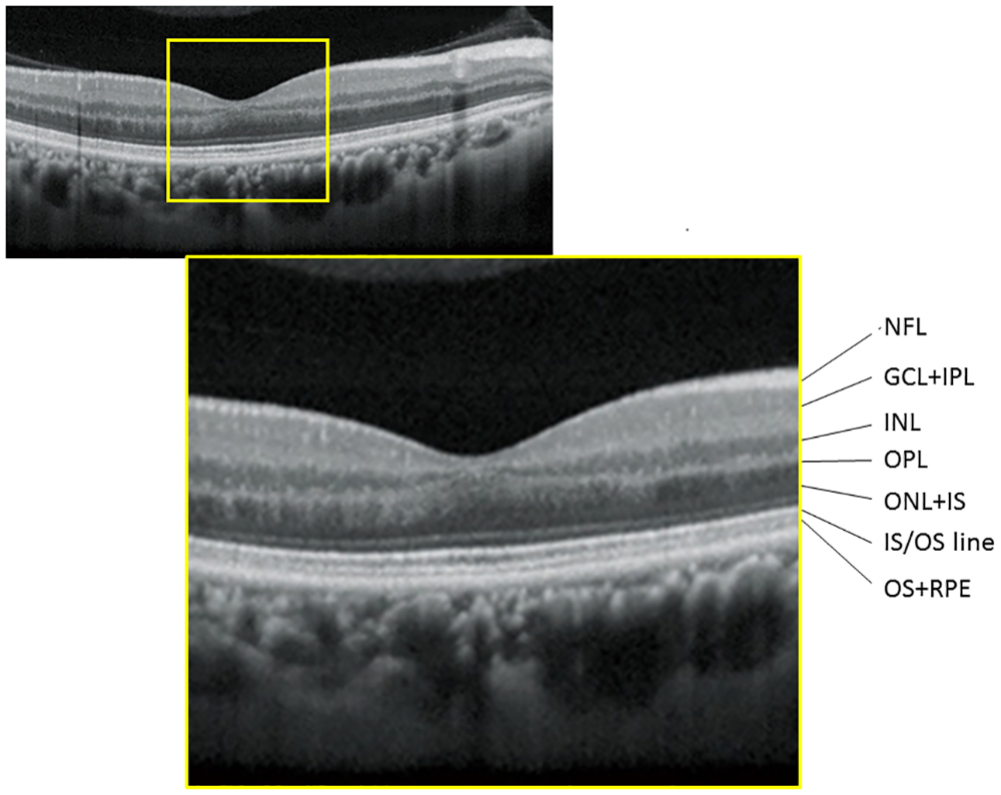
Macular retinal layer thickness measurements in a sample case. NFL: nerve fiber layer, GCL+IPL: ganglion cell and inner plexiform layer, INL: inner nuclear layer, OPL: outer plexiform layer, ONL+IS: outer nuclear layer and inner segment layer: OS+RPE outer segment layer and retinal pigment epithelium.

Similar to our previous report analyzing OCT data,[40] the fovea was automatically identified as the pixel with the thinnest retinal thickness close to the fixation point, and a square imaging area (9 × 9 mm) was centered on the fovea, excluding the area of the optic disc and parapapillary atrophy. Using software supplied from the manufacturer, thicknesses of: i) NFL, ii) GCL+IPL, iii) INL+OPL, iv) ONL+IS and v) OS+RPE (see Fig 1) were exported. These thicknesses were exported as a pixel image (512 × 128 pixels), and the mean thickness values of the whole analysis area (9.0 × 9.0 mm, corrected with axial length), excluding the optic disc and parapapillary atrophy, were calculated. Left eyes were mirror-imaged to a right eye orientation.

The structure-function relationship between mTH and each of the five retinal layer thickness measurements (NFL, GCL+IPL, INL+OPL, ONL+IS and OS+RPE) was analyzed using a linear model, with and without age, gender and axial length. The structure-function relationship was also investigated in a linear model with all five retinal layer thickness measurements included. In addition, the relationship between the thickness of the OS+RPE layer with the thickness measurements of the other layers (NFL, GCL+IPL, INL+OPL and ONL+IS) was also analyzed using a linear model. Finally, the influence of OS+RPE on mTH was investigated using partial correlation, which is a method to express the specific portion of variance explained by eliminating the effect of other variables (namely, NFL, GCL+IPL, INL+OPL, ONL+IS, age, gender and axial length) when assessing the correlation between two variables.[41–43]

All statistical analyses were carried out using the statistical programming language R (ver. 3.1.3, The R Foundation for Statistical Computing, Vienna, Austria).

## Results

Subject characteristics are given in Table 1. Fig 1 represents the layers of NFL, GCL+IPL, INL+OPL and OS+RPE in a sample case.

**Table 1:** Subjects demographics and comparison of variables between groups.

	Mean±SD	[range]
age, y.o.	61.5±10.1	[28.0 to 79.0]
axial length, mm	25.0±0.52	[24.0 to 26.0]
gender, male:female	51:67	
eye, right:left	58:60	

SD: standard deviation

**Fig 2:**
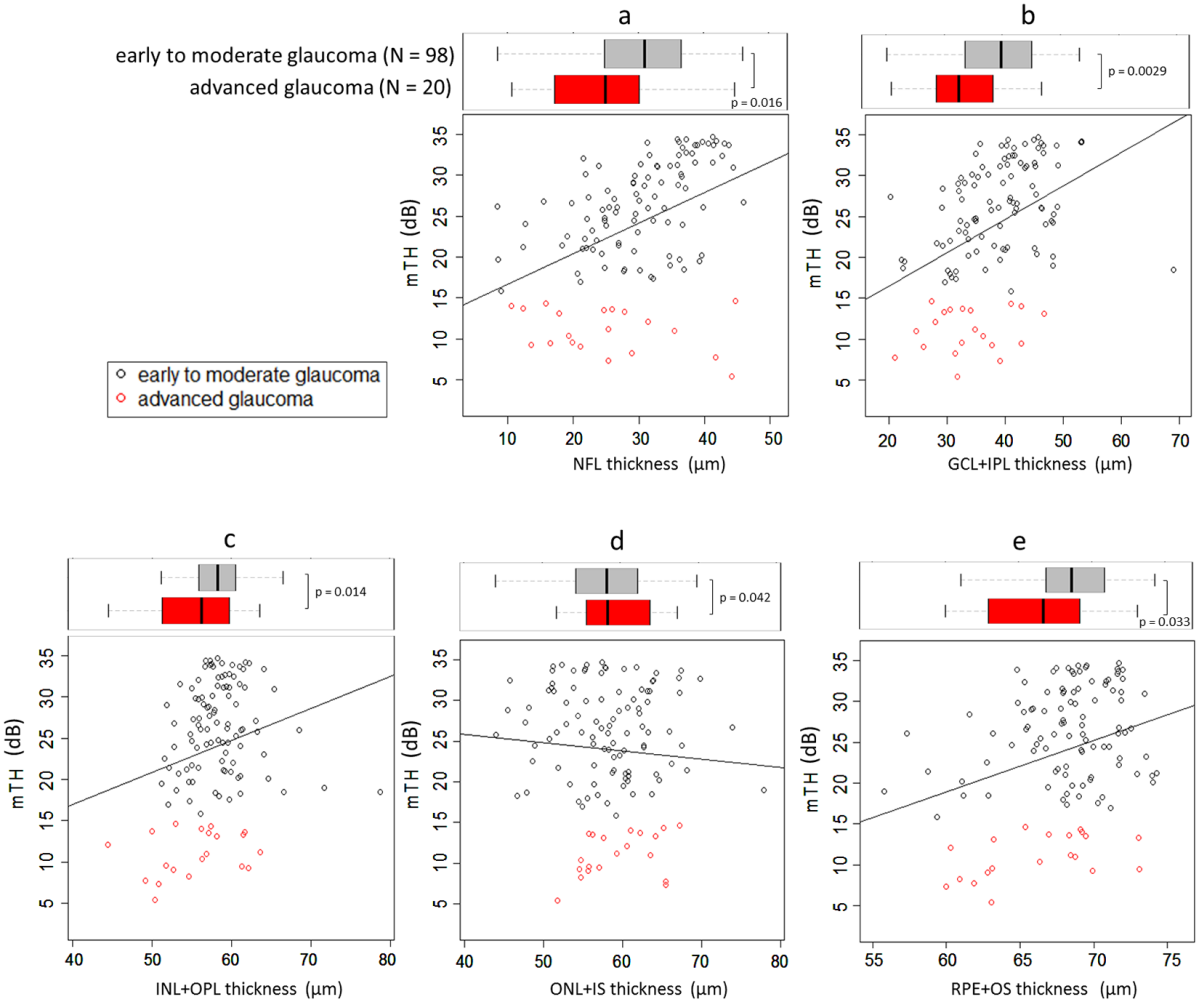
The relationship between retinal layer thickness measurements and mTH. The relationship between retinal layer thickness measurements and mTH was investigated. a: NFL (coefficient = 0.37, p <0.001), b: GCL+IPL (coefficient = 0.41, p <0.001), c: INL+OPL (coefficient = 0.39, p = 0.015) and e: OS+RPE (coefficient = 0.63, p <0.001) were significantly related to mTH however, a significant relationship was not observed between d: ONL+IS and mTH (coefficient = -0.10, p = 0.41). mTH: mean threshold, NFL: nerve fiber layer, GCL+IPL: ganglion cell and inner plexiform layer, INL+OPL: inner nuclear layer and outer plexiform layer, ONL+IS: outer nuclear layer and inner segment layer: OS+RPE outer segment layer and retinal pigment epithelium.

As shown in Fig 2, NFL (coefficient = 0.37, p <0.001), GCL+IPL (coefficient = 0.41, p <0.001), INL+OPL (coefficient = 0.39, p = 0.015) and OS+RPE (coefficient = 0.63, p <0.001) were significantly related to mTH in linear models without adjustment for age, gender and axial length, however, a significant relationship was not observed between ONL+IS and mTH (coefficient = -0.10, p = 0.41). In linear models—with adjustment for age, gender and axial length—NFL (coefficient = 0.33, p <0.001), GCL+IPL (coefficient = 0.42, p <0.001), INL+OPL (coefficient = 0.34, p = 0.0031), OS+RPE (coefficient = 0.63 p <0.001) remained significantly related to mTH, and, once again, no significant relationship was observed with ONL+IS (coefficient = -0.15, p = 0.22): see Table 2. Table 3 shows the linear model results from including all five retinal layer thicknesses as predictors: NFL (coefficient = 0.38, p <0.001), GCL+IPL (coefficient = 0.43, p <0.001) and OS+RPE (coefficient = 0.50, p = 0.011) were significantly decreased with the decrease of mTH. ONL+IS was significantly increased with the decrease of mTH (coefficient = -0.19, p = 0.031).

**Table 2:** The relationship between retinal layer thickness measurements and mTH (each thickness was analyzed in a separate linear model).

	coefficient	SE	p value		coefficient	SE	p value		coefficient	SE	p value
NFL, μm	0.33	0.075	<0.001	GCL+IPL, μm	0.42	0.080	<0.001	INL+OPL, μm	0.34	0.16	0.031
age, y.o.	-0.14	0.067	0.045	age, y.o.	-0.22	0.063	<0.001	age, y.o.	-0.20	0.069	0.0054
gender (for male)	0.70	1.31	0.59	gender (for male)	1.60	1.27	0.21	gender (for male)	1.40	1.39	0.32
axial length, mm	-0.45	1.2	0.72	axial length, mm	0.21	1.20	0.87	axial length, mm	-0.032	1.33	0.98
	coefficient	SE	p value		coefficient	SE	p value				
ONL+IS, μm	-0.15	0.12	0.22	OS+RPE, μm	0.50	0.19	0.011				
age, y.o.	-0.23	0.070	0.0017	age, y.o.	-0.15	0.072	0.039				
gender (for male)	1.57	1.43	0.28	gender (for male)	1.32	1.38	0.34				
axial length, mm	-0.61	1.33	0.65	axial length, mm	-0.14	0.31	0.91				

SE: standard error, NFL nerve fiber layer, GCL+IPL: ganglion cell and inner plexiform layer, INL+OPL: inner nuclear layer and outer plexiform layer, ONL+IS: outer nuclear layer and inner segment layer: OS+RPE outer segment layer and retinal pigment epithelium.

**Table 3:** The relationship between retinal layer thickness measurements and mTH (all thickness measurements were analyzed in the same linear model).

	coefficient	SE	p value
NFL, μm	0.38	0.056	<0.001
GCL+IPL, μm	0.43	0.070	<0.001
INL+OPL, μm	-0.17	0.15	0.26
ONL+IS, μm	-0.19	0.085	0.031
OS+RPE, μm	0.49	0.15	<0.001
age, y.o.	-0.085	0.052	0.11
gender (for male)	1.09	1.03	0.29
axial length, mm	0.46	0.64	0.47

mTH: mean threshold, SE: standard error, NFL: nerve fiber layer, GCL+IPL: ganglion cell and inner plexiform layer, INL+OPL: inner nuclear layer and outer plexiform layer, ONL+IS: outer nuclear layer and inner segment layer: OS+RPE outer segment layer and retinal pigment epithelium.

Partial correlation analysis suggested OS+RPE thickness is significantly (coefficient = 0.31, p <0.001) related to mTH, independent from NFL, GCL+IPL, INL+OPL, ONL+IS, age, gender and axial length.

As shown in Table 4, OS+RPE thickness was significantly related to age (coefficient = -0.13, p <0.001), but not to gender (p = 0.77) or axial length (p = 0.22).

**Table 4:** The relationship between RPE+OS thickness and age, axial length and gender.

	coefficient	SE	p value
age	-0.13	0.033	<0.001
gender (for male)	-0.19	0.66	0.77
axial length	-0.77	0.63	0.22

SE: standard error.

## Discussion

In the current study, OCT and HFA 10–2 VF measurements were carried out in glaucomatous eyes. Linear modelling revealed a significant relationship between OCT-measured RPE+OS thickness and mean 10–2 VF sensitivity, independent from the thicknesses of other retinal layers of NFL, GCL+IPL, INL+OPL, ONL+IS, as well as age, gender and axial length. Partial correlation analysis suggested OS+RPE thickness is significantly related to mTH, independent from NFL, GCL+IPL, INL+OPL, ONL+IS, age, gender and axial length.

Numerous previous studies have reported a decrease in inner retinal layer thickness, including the NFL and the GCL+IPL layers, in glaucoma patients.[32, 44–52] Indeed these measurements are now frequently used to diagnose glaucoma and measure progression of the disease. Most of these studies investigated the relationship between inner retinal layer thickness and VF sensitivity using the 24–2 or 30–2 HFA test patterns; however, the macular scanning region of OCT corresponds to a much narrower area of the VF, making the 10–2 HFA test pattern more relevant for investigating the structure-function relationship[37] yet fewer reports have investigated this central VF region.[53–58] In the current study, the relationship between NFL and GCL+IPL thicknesses against 10–2 HFA VF sensitivity was studied and, in agreement with previous reports, these layers were significantly related to the deterioration of VF sensitivity in the 10–2 VF.

In contrast, a change in thickness of PhR in glaucoma has not been investigated in detail. A limited number of studies have suggested an involvement of this layer in glaucoma,[29, 30] and other studies have produced contradicting results.[31–34] To the best of our knowledge, this is the first study to perform a detailed investigation of both OCT measurements and 10–2 HFA VF results in a large number of glaucomatous eyes. We observed that the RPE+OS layer thickness was significantly correlated with the visual sensitivity in glaucoma, as suggested by the correlation to RPE+OS thickness with and without other retinal layer thickness measurements. Furthermore, the results with the partial correlation suggested that thin REE+OS layer is disadvantageous for visual sensitivity, independent from the thickness of other layers.

The thickness of the RPE+OS layer may[59] or may not decrease[60] with an elongation of eye. The current study included only eyes with a narrow range of axial length (24 to 26 mm). In this population, we failed to observe a significant association between axial length and the thickness of the RPE+OS layer. The thickness of the RPE+OS layer, however, is known to increase with age.[61] The age of participants in our study ranged from 28 years old to 79 years old. The relationship between RPE+OS thickness and mTH was influenced by age (Table 2), however, a decrease in RPE+OS thickness with the advancement of glaucoma was evident even after adjusting for this ageing effect. Further, this age effect was not observed when all five thicknesses were analyzed together (Table 3).

A weak but significant negative relationship was observed between the thickness of the ONL+IS layer and mTH when other retinal layer thickness measurements were simultaneously investigated. The reason for this negative relationship is not entirely clear, but it is possible that eyes with equivalent RPE+OS thickness but a thicker PhR layer may suggest the original (prior to the development of glaucoma) thickness of RPE+OS was thick and thinning of RPE+OS due to the development of glaucoma was more evident. In retinitis pigmentosa, atrophic change in the photoreceptor layer starts in the outer segment and the inner segment is less affected by the disease.[62] The disease mechanism of retinitis pigmentosa is entirely different from that of glaucoma, however, the current results suggest predominant atrophic change is observed in the outer segment of the photoreceptor layer and its inner segment is preserved even in late stage glaucoma. In addition, there are many glial cells in this layer. The glial cells are a supportive structure in the retina and these cells may be robust to glaucomatous change.

Most retinal ganglion cells form synapse in the lateral geniculate nucleus (LGN), and degeneration in the LGN of glaucomatous eyes has been reported. The association between the RPE+OS layer thickness and visual sensitivity may be ascribed to the similar retrograde shrinkage. On the other hand, the OS layer is connected to the GC layer via neural cells, such as bipolar cells. Bipolar cells mainly locate in the INL layer, but thinning of INL+ONL layer was not observed in the current study. In addition, the thickness of the RPE+OS layer was significantly related to the thickness of the NFL, but not significantly related to GCL+IPL, in the current study. Another possible reason for the association between the RPE+OS layer thickness and visual sensitivity is that an eye who originally had a thick RPE+OS layer has a high visual sensitivity. For instance, Araie et al. have suggested that a thick macular GCC layer is associated with higher visual sensitivity in the corresponding macular region in normal eyes.[63] It is not entirely clear whether thick RPE+OS layer is associated with high visual sensitivity in normal eyes, but if this is the case, the current result could be attributed to this effect. A future study is needed to shed light on this issue.

One limitation of the current study is the lack of eyes with long or short axial lengths. A future study should be carried out to investigate RPE+OS thickness in myopic glaucoma patients, in particular. This will also provide us information about the predictive potential of this RPE+OS layer measurement to detect progression in glaucoma patients. Also, the segmentation software used in RS3000 has been validated by the manufacturer (by optical Jigs). It has also already been approved by Food and Drug Administration (FDA), comparing with RTVue-100 (Optovue Inc., CA, USA) for the clinical agreement, however not validated in clinical studies.

## Conclusion

In conclusion, the current study suggested RPE+OS thickness is associated with the visual sensitivity in glaucoma. The precise mechanism for this association is not clear, and further investigations are needed to shed light on this phenomenon.

## Supporting information

S1 Table All dataset. The dataset contains data for all patients included in the study.
(CSV)
